# Modulation of mitochondrial function by extracellular acidosis in tumor cells and normal fibroblasts: Role of signaling pathways

**DOI:** 10.1016/j.neo.2024.100999

**Published:** 2024-04-16

**Authors:** Carmen Degitz, Sarah Reime, Christina-Marie Baumbach, Mandy Rauschner, Oliver Thews

**Affiliations:** Julius Bernstein Institute of Physiology, University of Halle-Wittenberg, Magdeburger Str. 6 (Saale), Halle, 06112, Germany

**Keywords:** Tumor acidosis, Mitochondrial respiration MAP kinases, PI3K, Mitochondrial structure

## Abstract

In many tumors pronounced extracellular acidosis resulting from glycolytic metabolism is found. Since several environmental stress factors affect the mitochondrial activity the aim of the study was to analyze the impact of acidosis on cellular oxygen consumption and which signaling pathways may be involved in the regulation.

In two tumor cell lines and normal fibroblasts cellular oxygen consumption rate (OCR) and mitochondrial function were measured after 3 h at pH 6.6. Besides the activation of ERK1/2, p38 and PI3K signaling in the cytosolic and mitochondrial compartment, the mitochondrial structure and proteins related to mitochondria fission were analyzed.

The acidic extracellular environment increased OCR in tumor cells but not in fibroblasts. In parallel, the mitochondrial membrane potential increased at low pH. In both tumor lines (but not in fibroblasts), the phosphorylation of ERK1/2 and PI3K/Akt was significantly increased, and both cascades were involved in OCR modulation. The activation of signaling pathways was located predominantly in the mitochondrial compartment of the cells. At low pH, the mitochondrial structure in tumor cells showed structural changes related to elongation whereas mitochondria fragmentation was reduced indicating mitochondria fusion. However, these morphological changes were not related to ERK1/2 or PI3K signaling.

Acidic stress seems to induce an increased oxygen consumption, which might further aggravate tumor hypoxia. Low pH also induces mitochondria fusion that is not mediated by ERK1/2 or PI3K signaling. The mechanism by which these signaling cascades modulate the respiratory activity of tumor cells needs further investigation.

## Highlights

• Acidic stress induced an increased mitochondrial oxygen consumption (OCR).
• ERK1/2 and PI3K/Akt signaling was activated by acidosis and modulated OCR.
• Acidosis increased the mitochondrial potential which was ERK1/2-dependent.
• Low pH induced an elongation of the mitochondria and reduced fragmentation.

## Graphical abstract

**Figure:**
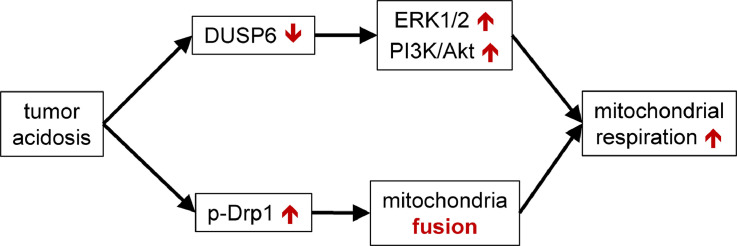

## Introduction

Many human and experimental tumors are characterized by an insufficient oxygen supply due to an inadequate perfusion. To maintain the energy demand, tumor cells can switch to glycolytic metabolism resulting in pronounced lactic acid production, which then leads to an acidification of the extracellular space [1]. However, even under conditions of a sufficient O_2_ supply, tumor cells often change to glycolytic metabolism to generate equivalents of carbon units and NADPH for the synthesis of macromolecules during proliferation. The phenomenon that tumors use glycolysis even under aerobic conditions is called “Warburg effect” and has been studied intensively during the last decades [2]. For this reason, tumors can become acidic with tumor cell mitochondria being sufficiently supplied with O_2_. Furthermore, the mitochondrial activity, and by this the cellular oxygen consumption rate (OCR), is adapted to the current needs of the tissue, i.e. different metabolic or environmental conditions. Energetic or oxidative stress, nutrient depletion or hypoxia (and many more) may lead to an altered OCR [3], [4], [5]. The regulation of mitochondrial respiration is modulated by changes in the activity of relevant enzymes (e.g., respiratory chain) [6] or by altering mitochondrial gene expression [7]. Several signaling pathways including MAP kinases, Akt, PKA or the PTEN-induced kinase 1 (PINK1) were shown to directly modulate mitochondrial activity [8]. Mitochondrial gene expression may be regulated by post-translational modifications of mtDNA, transcription factors, epigenetic modifications to mtDNA or nuclear transcription factors. Finally, mitochondria are able to sense metabolic conditions and by this regulate gene expression [9]. On the other hand, cellular respiration is altered by changes of the structure of the mitochondrial network. Fission and fusion of mitochondria in response to metabolic or environmental stress conditions are important mechanisms of cellular adaptation [10].

Besides hypoxia and oxidative stress, the acidic extracellular environment may also affect mitochondrial function. Acidosis was shown to modulate fatty acid metabolism and to reduce the activity of the complex I of the respiratory chain by hyperacetylation of proteins [11]. Hypercapnic acidosis (but not normocapnic acidosis) reduced the OCR significantly [12]. On the other hand, acidosis was also demonstrated to increase the cellular O_2_ consumption by upregulating the mitochondrial CYP24A1 expression [13]. It was postulated that acidosis inhibits mitochondria fission induced by hypoxia, which then leads to a higher mitochondrial activity during hypoxic stress [14]. This might also explain the finding that lactic acidosis induces aerobic respiration in order to maintain energy generation [15].

Since the aforementioned findings on the impact of acidosis on cellular respiration are somehow contradictory, the aim of the present study was to systematically study the impact of extra- and intracellular acidosis on mitochondrial respiration in two tumor and one normal cell line (i.e. fibroblasts, which are also part of the tumor tissue). To analyze the consequences of an altered respiration, the mitochondrial membrane potential was measured. It was also studied which signaling pathways are involved in the regulation of mitochondrial activity. To clarify possible mechanisms by which acidosis affects respiration, the structure of the mitochondrial network was analyzed, i.e. processes of mitochondria fission or fusion were quantified.

## Materials and methods

### Cell lines

The experiments were performed in two tumor cell lines: (1) subline AT1 of the Dunning rat prostate carcinoma R3327 (CLS # 500121, CLS GmbH, Eppelheim, Germany) and (2) human NCI-H358 bronchioalveolar carcinoma cells (ATCC #CRL-5807). The AT1 line is undifferentiated whereas NCI-H358 cells are weakly differentiated with glandular features. For comparison, normal rat kidney fibroblasts (NRK-49F (NRKF), ATCC #CRL-1570) were used. AT1 and NCI-H358 cells were cultured in RPMI medium supplemented with 10 % fetal calf serum (FCS) and NRK-49F cells in DMEM medium supplemented with 5 % FCS, respectively. Cells were kept at 37°C in a humidified 5 % CO_2_ atmosphere and were sub-cultivated twice per week. For the experiments, cells were kept in FCS-lacking medium for 24 h at normal pH (pH 7.4). The control pH of 7.4 and extracellular acidosis (pH 6.6) were obtained by buffering the media with NaHCO_3_, 10 mM HEPES and 10 mM MES (morpholinoethanesulfonic acid), and pH adjustment with 1 N NaOH.

## Chemicals

### If not stated otherwise, chemicals were purchased from Merck, Darmstadt, Germany

#### Signaling inhibitors and intracellular acidification

For studying the different signaling pathways, cells were incubated with inhibitors of p38 (SB203580, 10 µM), ERK1/2 (U0126, 10 µM) and Akt (Ly294002, 10 µM, Selleck Chemicals, Houston, USA) for 3 h. All inhibitors were dissolved in DMSO (which also served as control). To solely acidify the intracellular space (with constant extracellular pH 7.4) for 3 h, 40 mM NaCl were replaced by 40 mM propionic acid, a maneuver that was shown to acidify the intracellular compartment [16].

### Mitochondrial O_2_ consumption

The mitochondrial OCR was measured using the Seahorse XFe96 Analyzer (Agilent, Santa Clara CA, USA). This system measures fluoro-optically the O_2_ content in sealed wells of a 96-well plate. From the decrease over time, the oxygen consumption was calculated. The measurements were performed with the Cell Mito Stress Test Kit (Agilent) and the appropriate protocol. In this assay, the oxygen consumption was measured after incubation with various modulators of mitochondrial function. In brief, starting from the level of basal respiration oligomycin (1 µM) was added which inhibits ATP synthase so that the difference to basal respiration reflects O_2_ consumption for ATP synthesis. Then FCCP (2 µM) was added to uncouple the respiratory chain which results in a maximum OCR. Finally, rotenone and antimycin A (0.5 µM) were added to inhibit complexes I and III of the respiratory chain resulting in a complete blocking of mitochondrial respiration. The OCR measured under these conditions reflects the non-mitochondrial oxygen consumption of the cell. Suppl. Fig. S1 shows the OCR profile during the different assay steps schematically, the interpretation of the measurements as well as derived (calculated) parameters. At the end of the complete OCR protocol, the cells were stained with Hoechst 33342 and the number of cells per well was counted. With this value, the average oxygen consumption per cell was calculated.

### Mitochondrial membrane potential

The mitochondrial membrane potential (Ψ_M_) was measured by fluorescence microscopy using the Ψ_M_-sensitive dye JC-1. Cells were grown in a 96-well plate for 24 h and then incubated at pH 6.6 or 7.4 with and without different inhibitors for 3 h. During the last 30 min, JC-1 (Invitrogen, Darmstadt, Germany) at a final concentration of 2 µg/ml was added. As monomers, JC-1 shows green fluorescence (∼525 nm) but with increasing membrane potential JC-1 monomers form J-aggregates with a shift to red fluorescence (∼590 nm). The ratio of green to red fluorescence is regarded as a direct measure of Ψ_M_, being independent from mitochondrial size, shape, and density. The measurement was performed on a Cytation 3 Cell Imaging Reader (BioTek, Winooski VT, USA) using a 10x objective. Green fluorescence (ex. 469 nm, em. 525 nm) and red fluorescence (ex. 531 nm, em. 593 nm) images were taken from each well and after automatic cell segmentation, the ratio red/green (aggregates/monomers) was determined for each cell. For each well the average ratio of 50-500 cells was calculated. Each measurement was performed in triplicate.

### Mitochondrial structure

The structure of mitochondria under different conditions was analyzed by confocal microscopy using the Operetta CLS High Content Analysis System (PerkinElmer, Waltham, MA, USA). Therefore, cells were grown in CellCarrier Ultra 96-well plates (PerkinElmer) for 24 h and then incubated for 3 h at pH 6.6 or 7.4 with and without different inhibitors. Afterwards, cells were fixed and permeabilized. For mitochondria staining, cells were incubated with an anti-TOM20 antibody (#42406, Cell Signaling, Danvers MA, USA) and a secondary Oregon Green 488 antibody. Cells were imaged using the Operetta CLS system with a 40x water-immersion objective and spinning-disk confocal technology. The images were analyzed at single-cell level using Harmony 4.8 software (PerkinElmer). After segmentation of the cells, the cell area and the fluorescence intensity (as a measure of mitochondria mass) were calculated. Furthermore, morphological properties of the mitochondrial structure were determined by SER (Saddle, Edge, Ridge) texture analysis (Suppl. Fig. S2). Mitochondria fission is characterized by an increase of “spots” or “holes” structures whereas higher values of “edges”, “saddles” or “ridges” indicate mitochondria fusion [17]. For each well, the average numerical value of each texture parameter of 500-2500 cells was calculated. Each measurement was repeated in triplicate.

### Mitochondria isolation

To analyze the impact of signaling molecules directly at the mitochondria, cytosolic and mitochondrial fractions were separated. Therefore, cells were suspended in a homogenizing buffer (10 mM Tris-HCl, 0.25 M sucrose, 0.2 mM EDTA, protease inhibitor cocktail) and treated in a Dounce type homogenizer. After centrifugation (100 g, 4 min), the supernatant was again centrifuged (1000 g, 10 min), this latter supernatant represented the cytosolic fraction. The pellet was resuspended in a lysis buffer (0.05 mM Tris-HCl, 0.15 NaCl, 1 % protease and phosphatase inhibitor, 1 mM Na-orthovanadate, 15 mM NaF). After sonification, the suspension was again centrifuged (12,000 g, 5 min) and the resulting supernatant represented the mitochondrial fraction.

### Western blot

Western blotting was performed according to standard protocols. In brief, cells were lysed (0.5 M Tris-HCl pH 6.8; 10 % SDS; 10 % 2-mercaptoethanol; 20 % glycerol; 0.01 % bromophenol blue), separated by sodium dodecyl sulfate polyacrylamide gel electrophoresis, and transferred to a nitrocellulose membrane. Subsequently, membranes were incubated with antibodies specific for DRP1 (#14647, Cell Signaling), pDRP1-Ser616 (#PA5-64821; Thermo Fisher Scientific, Waltham, MA, USA), pDRP1-Ser637 (#4867, Cell Signaling), ERK1/2 (#4696, Cell Signaling), pERK1/2 (#9101, Cell Signaling), Akt (#2920, Cell Signaling) and pAkt (#4060, Cell Signaling). The bound primary antibody was visualized by IRDye secondary antibodies (Licor Biosciences, Lincoln, NE, USA) using the imaging system Odyssey (Licor Biosciences, Lincoln, NE, USA). Quantitative analysis was performed with Image Studio Lite software (Licor Biosciences).

### mRNA expression

For mRNA expression analyses, total RNA was isolated from cells using TRIzol (Thermo Fisher Scientific) according to the manufacturer's instructions. For qPCR validation, 1 µg RNA was subjected to reverse transcription with SuperScript II reverse transcriptase (Thermo Fisher Scientific) and analyzed by qPCR using the Platinum SYBR Green qPCR Supermix (Thermo Fisher Scientific). The obtained data were normalized against Hprt1 and were related to the respective control. Suppl. Tab. S1 shows the primers used.

### Metabolic parameters

Glucose and lactate concentrations in the media were determined enzymatically. For glucose the hexokinase+glucose-6-phosphat dehydrogenase and for lactate the lactate dehydrogenase reaction were used. Glutamine concentration was determined using the EnzyChrom Glutamine Assay Kit (BioAssay Systems, Hayward, USA) according to the manufacturer's instructions. Cellular ATP levels were measured using a bioluminescence reaction (ATP Bioluminescence Assay Kit CLS II, Roche, Mannheim, Germany), the concentration was measured in lysed cells according to the manufacturer's instructions. All measurements were normalized to total cell protein.

### Statistical analysis

Results are expressed as means±SEM. Differences between groups were assessed by the two-tailed t-test for paired and unpaired samples, respectively. The significance level was set at α=5 % for all comparisons.

## Results

### Extracellular acidification can increase cellular respiration

**Fig. 1:**
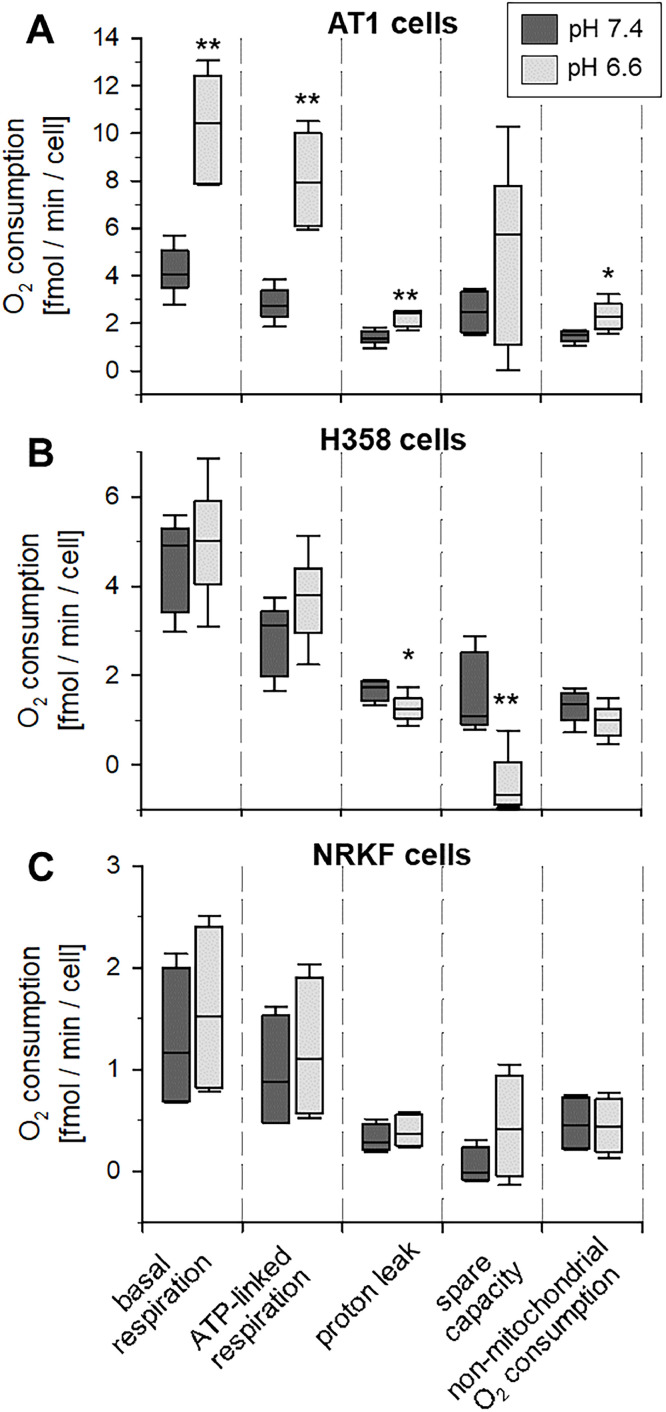
Impact of the extracellular pH on cellular respiration of (**A)** AT1 and (**B**) H358 tumor cells as well as in (**C**) normal fibroblasts (NRKF) after 3 h incubation. *n* = 4-6, (*) *p* < 0.05, (**) *p* < 0.01 pH 6.6 *vs*. 7.4.

For measurements of the impact of the extracellular pH on OCR, tumor cells and fibroblasts were incubated at pH 6.6 or 7.4 for 3 h. Fig. 1 shows the oxygen consumption of the different parameters of respiration (as described in Suppl. Fig. S1) in AT1, H358 and NRKF cells. The basal O_2_ consumption in both tumor lines was approx. 3.5-fold higher than in fibroblasts. Under acidic conditions, AT1 tumor cells showed more than a doubling of basal and ATP-linked respiration and a slight increase in proton leak and non-mitochondrial consumption. H358 tumor cells showed an increase (not statistically significant) of ATP-linked OCR by 30 %. All other values were comparable at pH 6.6 and 7.4. Only the spare capacity (after uncoupling the respiratory chain with FCCP) was reduced to almost 0 fmol/min/cell indicating that during acidosis the O_2_ consumption was almost at its maximum even under basal conditions. In normal fibroblasts, OCR did not change regarding the extracellular pH. The basal and ATP-linked respiration were only marginally increased by the extracellular pH.

In AT1 cells, the increased OCR was associated with a slightly higher glucose consumption whereas the lactate production remained unchanged (Suppl. Fig. S3). Therefore, the ratio of produced lactate per consumed glucose was significantly reduced under acidic conditions. Low pH also led to a slightly reduced glutamine consumption (Suppl. Fig. S3). Even though the mitochondrial activity and glucose consumption were increased under acidic conditions, the cellular ATP level remained almost constant (Suppl. Fig. S4).

### pH-dependent ERK1/2 and PI3K phosphorylation is located at the mitochondria and modulates respiration

**Fig. 2:**
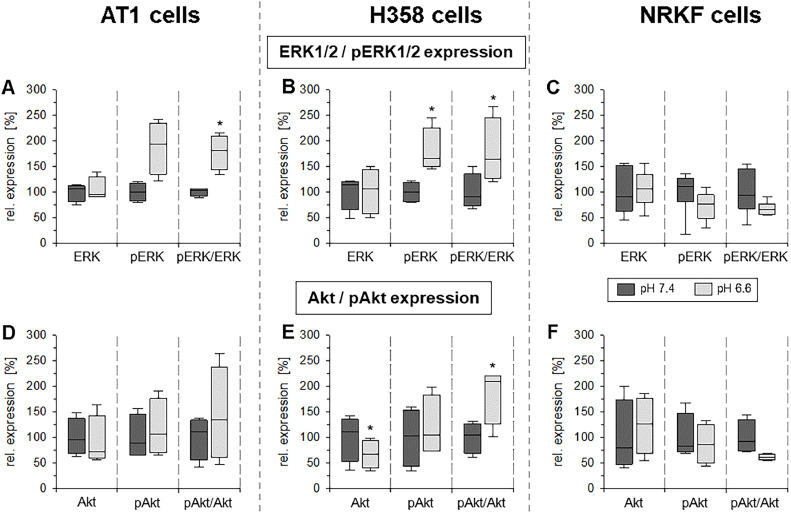
Impact of the extracellular pH (pH 6.6 and 7.4 for 3 h) on ERK1/2 and Akt phosphorylation in AT1 (**A**+**D**) and H358 (**B**+**E**) tumor cells and NRKF fibroblasts (**C**+**F**). *n* = 4-6, (*) *p* < 0.05 pH 6.6 *vs*. 7.4.

Since previous studies already indicated that extracellular acidosis can activate signaling pathways [18,19], it was tested whether ERK1/2 or PI3K/Akt were activated under acidic conditions in the three cell lines of the present study. Fig. 2 shows the expression of ERK1/2 and Akt as well as their phosphorylation after 3 h at pH 6.6. In both tumor lines (AT1, H358) but not in the normal fibroblasts (NRKF), ERK1/2 phosphorylation was significantly increased. The expression of total ERK1/2 was not altered in all cell lines. Akt phosphorylation was increased in H358 cells, and a slight trend was seen in AT1 cells, whereas in NRKF cells Akt expression and phosphorylation status were independent from the pH. With these results it was further analyzed whether the changes in OCR may result from the pH-dependent activation of p38, ERK1/2 or PI3K.

**Fig. 3:**
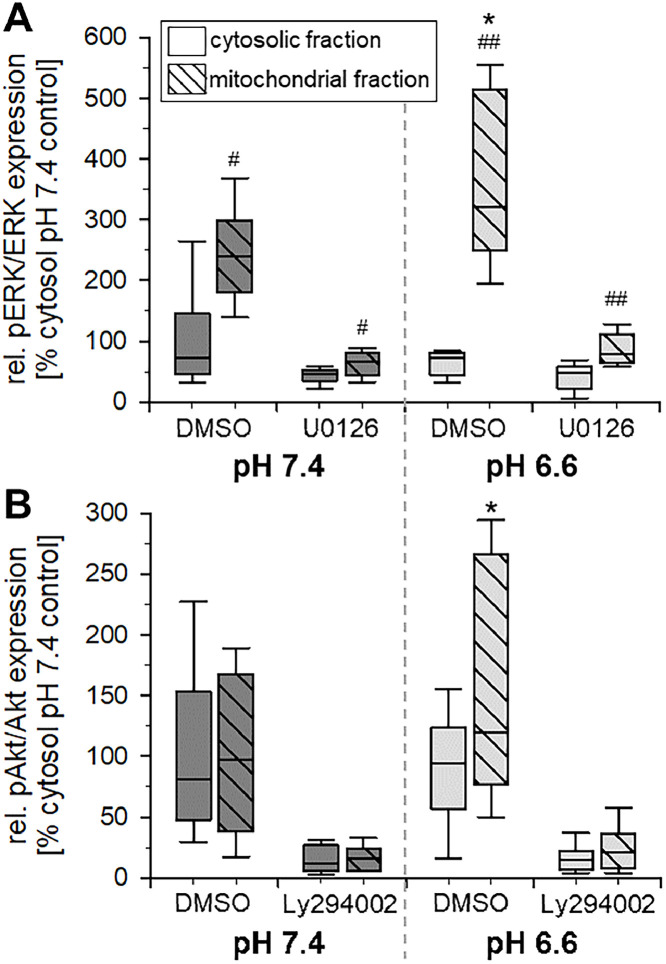
Impact of the extracellular pH (6.6 and 7.4 for 3 h) on the phosphorylation of (**A**) ERK1/2 and (**B**) Akt kinases in the cytosolic and the mitochondrial cell fraction of AT1 tumor cells. Treatment of the cells with inhibitors of ERK1/2 (U0126) or PI3K (Ly294002) served as negative controls. *n* = 6, (*) *p* < 0.05 pH 6.6 *vs*. 7.4; (#) *p* < 0.05, (##) *p* < 0.01 cytosolic *vs*. mitochondrial fraction.

Since it has been described that phosphorylation of signaling factors can be located directly at the mitochondria and by this may influence mitochondrial respiration [8,20,21], it was analyzed whether ERK1/2 and Akt phosphorylation can be detected at the mitochondria. Therefore, cytosolic and mitochondrial fractions of AT1 cells were separated and ERK1/2 as well as Akt expression and phosphorylation were measured. Fig. 3 and Suppl. Fig. S5 show the impact of acidosis on the expression and phosphorylation of ERK1/2 and Akt in the mitochondrial and cytosolic fraction, respectively. Interestingly, the increase in ERK1/2 phosphorylation and (to a lower extent) of Akt took place at the mitochondria whereas in the cytosol almost no change was seen under acidic conditions. It can be concluded that the main part of the ERK1/2 and Akt phosphorylation seen in whole cell lysates (Fig. 2) originates from the mitochondria (Fig. 3).

**Fig. 4:**
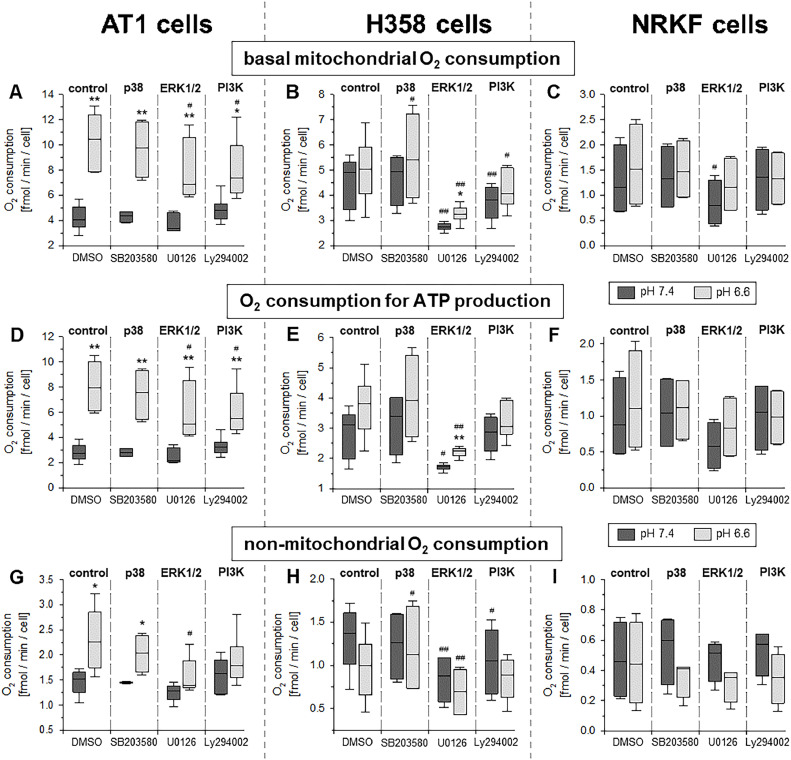
Parameters of cellular respiration after 3 h incubation at pH 6.6 or 7.4 and concomitant application of inhibitors of the p38 (SB203580), ERK1/2 (U0126) and PI3K (LY294002) kinases in AT1 (**A, D, G**) and H358 (**B, E, H**) tumor cells and NRKF fibroblasts (**C, F, I**). *n* = 4-6, (*) *p* < 0.05, (**) *p* < 0.01 pH 6.6 *vs*. 7.4; (#) *p* < 0.05, (##) *p* < 0.01 *vs*. respective control (DMSO).

Fig. 4 shows the respiration parameters (basal, ATP-linked and non-mitochondrial O_2_ consumption) at pH 6.6 and 7.4 in combination with inhibition of p38 (with SB203580), ERK1/2 (with U0126) or PI3K (with LY294002) kinases. In AT1 and H358 cells, inhibition of ERK1/2 and PI3K both led to a significant decrease of the basal and the ATP-linked O_2_ consumption. However, the control level at pH 7.4 was not reached which may indicate that the increase of respiration under acidic condition is only partially mediated by these signaling pathways. In H358 cells, ERK1/2 and PI3K inhibition also reduced the basal respiration at pH 7.4 indicating that these signaling pathways play a more general role in mitochondrial function. However, the non-mitochondrial O_2_ consumption in both tumor lines was reduced by ERK1/2 and PI3K inhibition as well. Nevertheless, even though the ATP-linked OCR of AT1 cells was modulated by inhibitors of the different signaling pathways (Fig. 4D), the cellular ATP level remained unchanged at both pH (Suppl. Fig. S4). In normal fibroblasts, none of the signaling pathways had a marked influence on OCR neither at pH 6.6 nor 7.4.

### Intracellular acidification also increases OCR slightly mediated by MAPK and PI3K

**Fig. 5:**
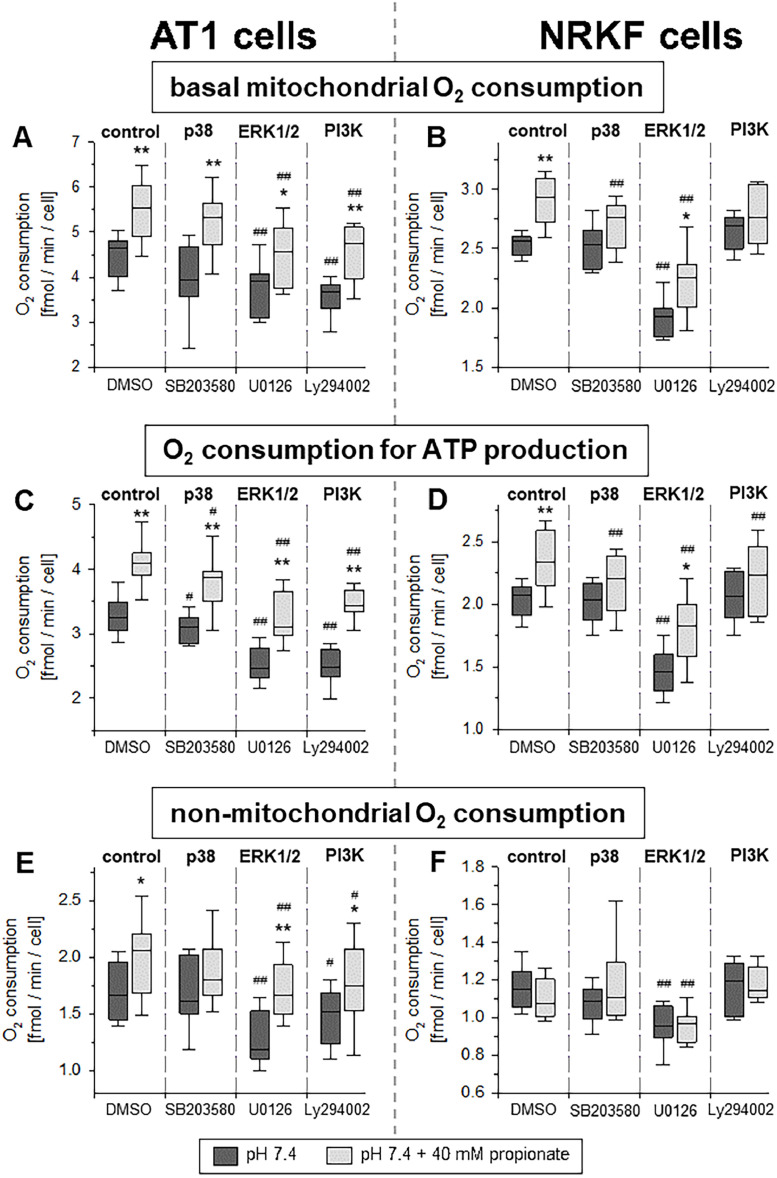
Impact of sole intracellular acidification by replacing 40 mM NaCl with 40 mM propionic acid on cellular respiration. Parameters of OCR after 3 h incubation at pH 7.4 with or without propionate and concomitant application of inhibitors of the p38 (SB203580), ERK1/2 (U0126) and PI3K (LY294002) kinases in AT1 (**A, C, E**) tumor cells and NRKF fibroblasts (**B, D, F**). *n* = 6-8, (*) *p* < 0.05, (**) *p* < 0.01 with *vs*. w/o propionate; (#) *p* < 0.05, (##) *p* < 0.01 *vs*. respective control (DMSO).

Previous experiments showed, firstly, that extracellular acidosis leads to the rapid acidification of the intracellular space [22] and, secondly, that the sole intracellular acidification can activate MAP kinases [23]. Therefore, further experiments were performed to analyze whether the sole reduction of the intracellular pH (with normal extracellular pH) also affects respiration. Cells were treated (at constant extracellular pH 7.4) with 40 mM propionic acid for 3 h, a method to acidify the intracellular compartment [16]. As shown in Fig. 5, in AT1 as well as in NRKF cells intracellular acidification slightly increased mitochondrial respiration. In AT1 cells, however, this effect was much smaller compared to that under extracellular acidosis (increase by 20-25 %). In contrast to extracellular acidosis (Fig. 4), the intracellular acidification in NRKF fibroblasts increased mitochondrial function significantly by approx. 20 %. The basal and ATP-linked respiration of NRKF cells was markedly reduced by inhibition of ERK1/2 signaling under control as well as under acidic conditions. The non-mitochondrial respiration in fibroblasts was independent from the intracellular pH but was also reduced by ERK1/2 inhibition.

### Mitochondrial potential is increased by acidosis and is ERK1/2-dependent

**Fig. 6:**
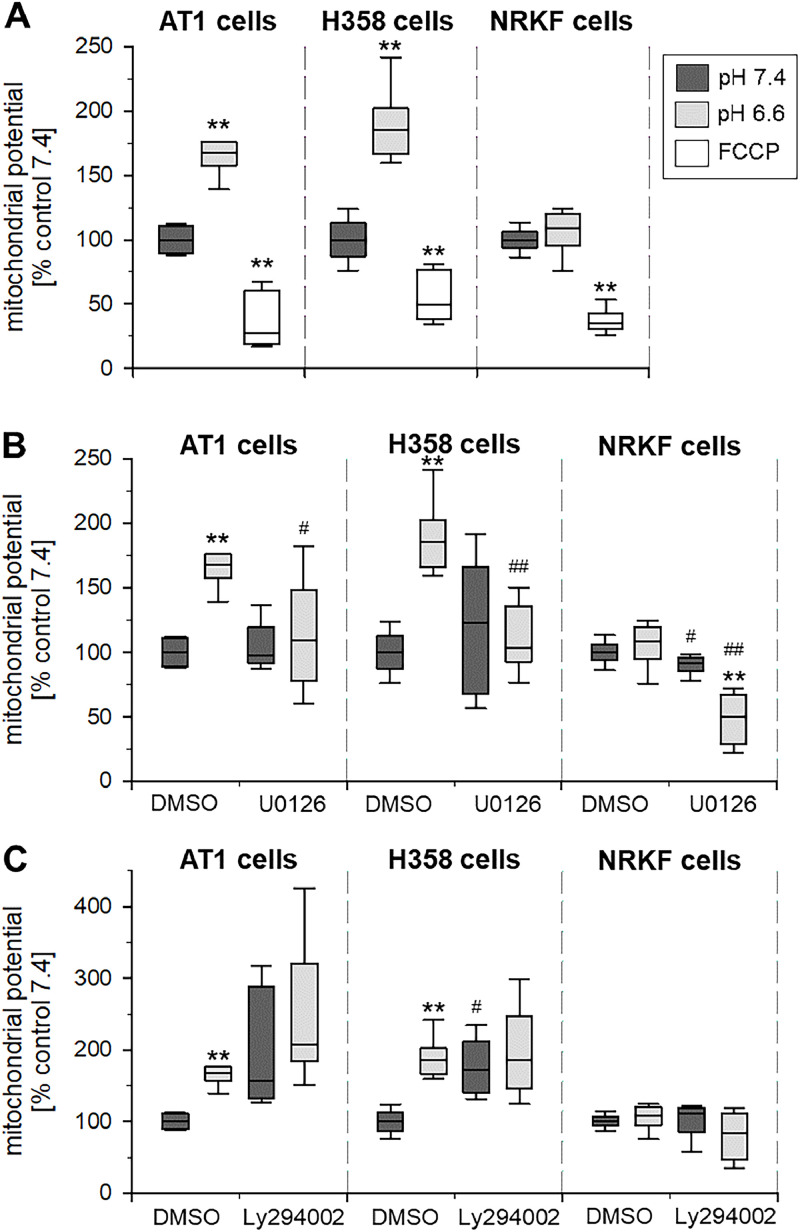
(**A**) Mitochondrial potential Ψ_M_ (measured by the cellular fluorescence of JC-1) in AT1 and H358 tumor cells and NRKF fibroblasts at pH 6.6 and 7.4 for 3 h (*n* = 6). Incubation with FCCP at pH 7.4 was used as negative control (breakdown of Ψ_M_ by uncoupling of the respiratory chain). Impact of additional inhibition of (**B**) ERK1/2 (U0126) and (**C**) PI3K (LY294002) on the mitochondrial potential. *n* = 5-6, (*) *p* < 0.05, (**) *p* < 0.01 pH 6.6 *vs*. 7.4; (#) *p* < 0.05, (##) *p* < 0.01 *vs*. respective control (DMSO).

Fig. 6A shows the mitochondrial potential (measured by the Ψ_M_-sensitive JC-1) in the three cell lines after 3 h incubation at pH 6.6 and 7.4. In both tumor lines, the potential increased significantly under acidic conditions whereas in NRKF cells Ψ_M_ was independent from the extracellular pH. In all three cell lines, inhibition of ERK1/2 signaling (by U0126) had a strong effect on the mitochondrial potential under acidic conditions (Fig. 6B). In both tumor lines, ERK1/2 inhibition reduced Ψ_M_ to the control level of cells at pH 7.4. In NRKF cells, where acidosis *per se* had no impact on the potential, ERK1/2 inhibition reduced Ψ_M_ slightly (but significantly) under both pH conditions. Inhibition of PI3K (by Ly294002) had only a minor impact on Ψ_M_ (Fig. 6C). Only under control conditions (pH 7.4), inhibition of PI3K led to an increase of the mitochondrial potential in both tumor lines.

### Acidosis leads to morphological changes of mitochondria

**Fig. 7:**
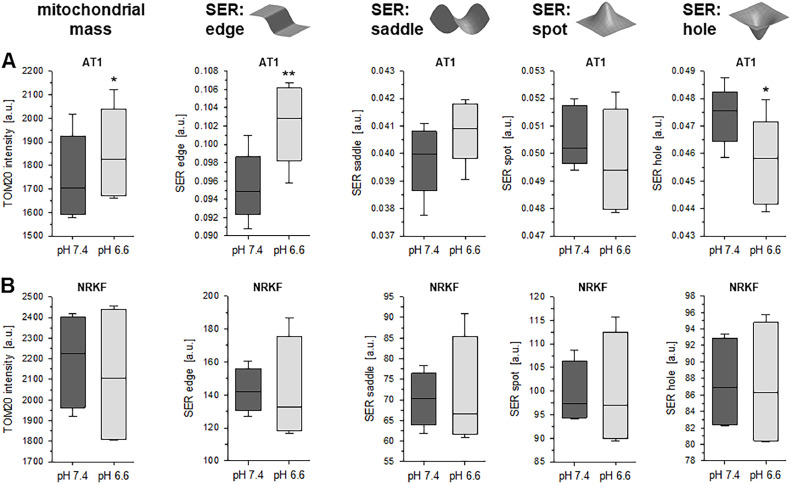
Impact of the extracellular pH (6.6 and 7.4) on the cellular mitochondrial mass and the mitochondrial morphology in (**A**) AT1 tumor cells and (**B**) NRKF fibroblasts. Mitochondrial structure was quantified using the SER (Saddle, Edge, Ridge) texture analysis of cells stained with the mitochondria marker TOM20. The sketches above the graphs show the schematic structures which were quantified by the respective SER parameter. *n* = 4-6, (*) *p* < 0.05, (**) *p* < 0.01 pH 6.6 *vs*. 7.4.

In order to analyze morphological changes of the mitochondria, TOM20 protein was stained. Using the immuno-fluorescence images, the mitochondrial structure was evaluated by SER (Saddle, Edge, Ridge) texture analysis (Suppl. Fig. S2). In addition, the total TOM20 fluorescence was used as an indicator of the mitochondrial mass within the cells. As shown in Fig. 7, the total mitochondrial mass was slightly increased in AT1 tumor cells during acidosis (Fig. 7A) whereas in NRKF cells the mass was unchanged (Fig. 7B). In AT1 tumor cells, the extent of linear structures significantly increased at low pH (Fig. 7A, SER: “edge”) whereas the number of isolated “holes” was significantly reduced. These characteristics indicate more elongated, continuous mitochondria, which may give evidence for mitochondria fusion [17]. In contrast, the structural parameters in NRKF fibroblasts did not change significantly at low pH (Fig. 7B). In further experiments, it was tested whether the acidosis-induced changes in mitochondrial structure were mediated by one of the signaling pathways (p38, ERK1/2, PI3K). The results showed that the pH dependency in AT1 cells was almost unaffected by treating the cells with p38 or PI3K inhibitors (Suppl. Fig. S6). Only incubating AT1 cells with the ERK1/2 inhibitor (U0126) reduced the “edge” parameter slightly and increased the “hole” parameter which might be an indication that the acidosis-induced mitochondria fusion was inhibited. In NRKF fibroblasts, none of the inhibitors had an effect on mitochondrial morphology (Suppl. Fig. S7).

**Fig. 8:**
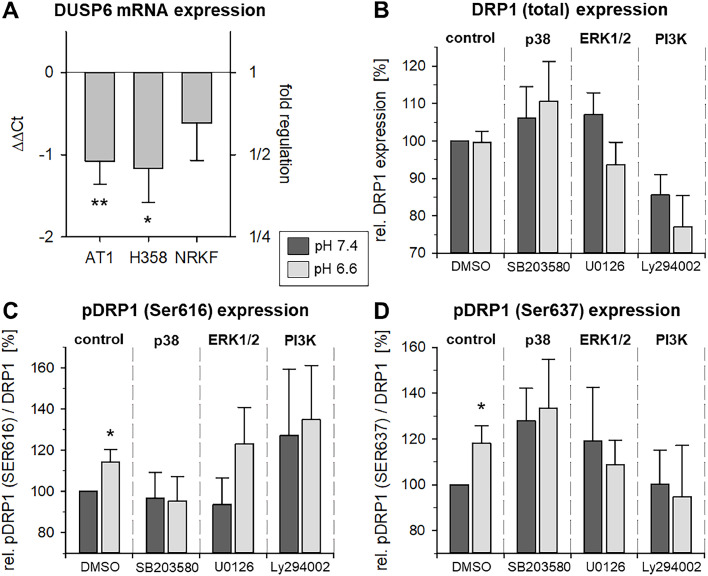
(**A**) mRNA expression of DUSP6 in the three cells lines (AT1, H358, NRKF) after incubation at pH 6.6. Relative expression of (**B**) total DRP1 protein, (**C**) phospho-DRP1 (Ser616) and (**D**) phospho-DRP1 (Ser637) in AT1 tumor cells after 3 h at pH 6.6 or 7.4 and concomitant inhibition of p38, ERK1/2 and PI3K signaling (with SB203580, U0126 or Ly294002). *n* = 4-10, (*) *p* < 0.05, (**) *p* < 0.01 pH 6.6 *vs*. 7.4.

Since it has been described that ERK1/2 activation promotes DRP1 (dynamin-related protein 1)-dependent mitochondrial fission in which the downregulation of the MAPK phosphatase DUSP6 (dual-specificity phosphatase 6) plays a role [24], DUSP6 expression as well as DRP1 phosphorylation were measured under acidic conditions. In both tumor lines, DUSP6 expression at mRNA level was significantly reduced after 3 h at pH 6.6 (Fig. 8A). In NRKF fibroblasts, the expression was also lowered but not significantly. The expression of total DRP1, which is a central activator of mitochondria fission after binding, showed under control conditions no pH-dependent differences in AT1 cells (Fig. 8B). Inhibition of p38 induced a slight increase in DRP1 expression (not significant). When inhibiting ERK1/2 or PI3K, the expression was slightly reduced under acidic conditions. The phosphorylation of DRP1 at different sites leads to fundamentally different consequences in terms of function. Phosphorylation at Ser616 promotes mitochondrial fission whereas phosphorylation at Ser637 detaches DRP1 from mitochondria and inhibits fission [25]. Under control conditions (DMSO), the phosphorylation at both sites was increased by about 20 % when cells were incubated at pH 6.6 (Fig. 8C+D). Inhibition of p38 led to a slightly higher phosphorylation at Ser637 (Fig. 8D) whereas PI3K inhibition resulted in a higher Ser616 phosphorylation (Fig. 8D). However, these changes were independent from the extracellular pH.

## Discussion

The energy metabolism in normal tissues as well as in tumors mainly depends on oxidative phosphorylation (OXPHOS) which is the major O_2_ consuming process of cells. Since the oxygenation status of the tissue results from the balance of O_2_ supply and consumption [26], the mitochondrial respiration is an important parameter determining the tissue pO_2_ and by this affecting the malignant potential and therapy resistance [27]. The mitochondrial activity is modulated by various factors of the metabolic microenvironment of tumors such as hypoxic or oxidative stress [3,5]. The results of the present study show that an acidic extracellular environment increases the cellular O_2_ consumption of tumor cells markedly, however, in normal fibroblast, the pH had almost no effect (Fig. 1). Similar results were found in glioma stem cells where the incubation at pH 6.8 increased the mitochondrial and the non-mitochondrial oxygen consumption [13]. In normal cortical neurons, Khacho et al. [14] showed that extracellular acidosis (pH 6.5) increased OCR by about 50 % but only after incubating the cells with FCCP (uncoupling the respiratory chain). ATP-linked and non-mitochondrial O_2_ consumption were unaffected by low pH. Similar results were described by Xie et al. [28] in a model of lactic acidosis. In their study, however, the lactate anion *per se* also had an impact on cellular metabolism leading to a reduced glucose consumption and lactate generation. This is in contrast to the present study in which acidosis increased glucose consumption resulting in a reduced lactate production/glucose consumption ratio (Suppl. Fig. S3).

Extracellular acidosis has been shown to modulate the activation state of different signaling cascades such as ERK1/2, p38 or PI3K/Akt/mTOR [18,19]. By analyzing the phosphorylation state of these pathways, it was shown that in both tumor lines (but not in normal fibroblasts) the phosphorylation of ERK1/2 and (at least partially) of PI3K/Akt was significantly increased (Fig. 2). The activation of these pathways was located preferentially in the mitochondrial rather than in the cytosolic fraction of the cells (Fig. 3). Such a direct interaction of different signaling factors at mitochondria and by this a regulation of the mitochondrial function in tumors and normal cells has been shown by others [8,20,[29], [30], [31]]. In the present study, inhibition of ERK1/2 and PI3K signaling reduced the mitochondrial as well as the non-mitochondrial oxygen consumption in both tumor lines but not in normal NRKF cells (Fig. 4). These results are in accordance with findings by others that ERK1/2 [29,32,33] and PI3K/Akt [8,34] are able to regulate mitochondrial oxidative phosphorylation. In the present experiments, the increased OCR by acidosis was counteracted by inhibiting signaling. From these results it might be concluded that acidosis leads (at least partially) to an increased mitochondrial function via ERK1/2 and/or PI3K but not by p38 signaling. In addition to O_2_ consumption also the mitochondrial membrane potential as an indicator of mitochondrial activity was significantly increased by acidosis, but the increase could be completely abolished by ERK1/2 inhibition supporting the aforementioned conclusions (Fig. 6). The principal role of ERK1/2 signaling for the membrane potential was also demonstrated in macrophages [35]. In contrast, PI3K signaling had almost no impact on the membrane potential even though OCR was depending on this signaling cascade.

Many mechanisms have been discussed by which the respiratory activity of mitochondria is adapted to the metabolic demand of the cell [36,37]. One important mechanism is the functional organization of the mitochondrial structure by fission and fusion [10]. Especially under stress conditions, mitochondria undergo both fission leading to mitophagy or fusion resulting in a higher energy production. The analysis of the mitochondrial structure under acidic conditions indicated that in tumor cells, low pH leads to structural changes related to elongation and reduced fragmentation. In AT1 cells, fission was reduced whereas in fibroblasts no marked changes were found (Fig. 7). An elongation of mitochondria at low pH was described for neural cells [14]. A central protein responsible for the regulation of fission is DRP1 [38,39], which itself is regulated by phosphorylation [25,40]. Several kinases and phosphatases were described to modulate the DRP1 phosphorylation status. ERK1/2 [24,41,42] but also PI3K/Akt/mTOR [43] can directly regulate the DRP1 phosphorylation but also indirectly by affecting the DUSP6 activity [24,44]. Since in the present study acidosis reduced the DUSP6 expression in both tumor lines (Fig. 8A), it might be possible that the acidosis effect on mitochondrial structure (and by this on OCR) was mediated by the DRP1 phosphorylation status. However, DRP1 has different phosphorylation sites which once phosphorylated either activate DRP1 (phospho-DRP1 Ser616) forcing fission or inhibit DRP1 (phospho-DRP1 Ser637) leading to fusion [25,40]. The present study showed that extracellular acidosis did not change the total expression of DRP1 (Fig. 8B). Surprisingly, the DRP1 phosphorylation at both sites was significantly increased by acidosis (Fig. 8C+D). In addition, the inhibition of ERK1/2 as well as PI3K signaling had no clear and consistent effect on the DRP1 phosphorylation.

## Conclusions

In conclusion, the present study demonstrates that an acidic extracellular environment, which is a common finding in tumors, increases the mitochondrial O_2_ consumption and by this the functional activity of mitochondria as indicated by the membrane potential. Here, the pH effect was not seen in fibroblasts which are also part of the tumor tissue. The increase of the OCR in tumor cells was mediated (at least partially) by ERK1/2 and PI3K/Akt signaling. In parallel, the morphological analysis indicated mitochondrial fusion, which could explain the higher OCR. However, these morphological changes were not related to the signaling pathways analyzed and could not be explained by an ERK1/2- or PI3K-induced DRP1 phosphorylation. Therefore, further studies need to analyze the role of other signaling cascades (PKA, AMPK, intracellular Ca^2+^) that are known to modulate DRP1 and might be pH dependent. On the other hand, the mechanisms by which acidosis-induced ERK1/2 and PI3K activation affects mitochondrial respiration independently from regulating fusion or fission have to be addressed in further studies. Since mitochondrial function plays a relevant role in the malignant progression of tumors, the knowledge of the impact of the acidic environment may provide therapeutic approaches to either counteract the development of acidosis or to interrupt the signaling pathways modulating cellular respiration.

## Availability of data and materials

The data presented in this study are available on request from the corresponding author.

## CRediT authorship contribution statement

**Carmen Degitz:** Conceptualization, Formal analysis, Investigation, Methodology, Writing – review & editing. **Sarah Reime:** Investigation. **Christina-Marie Baumbach:** Formal analysis, Investigation, Writing – review & editing. **Mandy Rauschner:** Formal analysis, Investigation, Writing – review & editing. **Oliver Thews:** Conceptualization, Formal analysis, Investigation, Methodology, Project administration, Resources, Visualization, Writing – original draft.

## Declaration of competing interest

The authors declare no competing conflicts of interests.

## Appendix Supplementary materials

Supplementary materials Supplementary material associated with this article can be found in the online version at doi:**XXXX. Tab. S1**: Primers used for qPCR. **Fig. S1**: Schematic diagram of the oxygen consumption measurements. **Fig. S2**: Intensity patterns analyzed by SER texture analysis. **Fig. S3**: Glucose, lactate and glutamine metabolism during acidosis. **Fig. S4**: Cellular ATP levels during acidosis and concomitant inhibition of signaling pathways. **Fig. S5**: Erk1/2 and Akt signaling during acidosis in the cytosolic and mitochondrial fraction of tumor cells. **Fig. S6**: Impact of signaling pathways on mitochondrial morphology and mass in AT1 tumor cells during acidosis. **Fig. S7**: Impact of signaling pathways on mitochondrial morphology and mass in normal fibroblasts during acidosis. Image, application 1
