# Supplementary material for: Modulation of mitochondrial function by extracellular acidosis in tumor cells and normal fibroblasts: Role of signaling pathways
Source: Neoplasia. 2024 Apr 16;52:100999. doi: 10.1016/j.neo.2024.100999 (PMC11036092; doi:10.1016/j.neo.2024.100999)
Supplement: Supplementary file 1 — Supplementary materials Supplementary material associated with this article can be found in the online version at doi:XXXX. Tab. S1: Primers used for qPCR. Fig. S1: Schematic diagram of the oxygen consumption measurements. Fig. S2: Intensity patterns analyzed by SER texture analysis. Fig. S3: Glucose, lactate and glutamine metabolism during acidosis. Fig. S4: Cellular ATP levels during acidosis and concomitant inhibition of signaling pathways. Fig. S5: Erk1/2 and Akt signaling during acidosis in the cytosolic and mitochondrial fraction of tumor cells. Fig. S6: Impact of signaling pathways on mitochondrial morphology and mass in AT1 tumor cells during acidosis. Fig. S7: Impact of signaling pathways on mitochondrial morphology and mass in normal fibroblasts during acidosis. [file mmc1.pdf]

# Modulation of mitochondrial function by extracellular acidosis in tumor cells and normal fibroblasts: role of signaling pathways

Carmen Degitz, Sarah Reime, Christina-Marie Baumbach, Mandy Rauschner, Oliver Thews
Institute of Physiology, University Halle, Magdeburger Str. 6, 06112 Halle (Saale), Germany

## Supplementary Material

**Table S1:** Primers used for quantitative PCR.

| target | forward primer | reverse primer |
|---|---|---|
| *Dusp6* | TGAGGATCAGCTCCGACTCT | TTTGCCTCGGGCTTCATCTAT |
| *Hprt1* | ACCAGTCAACGGGGGACATA | TTGGGGCTGTACTGCTTGAC |

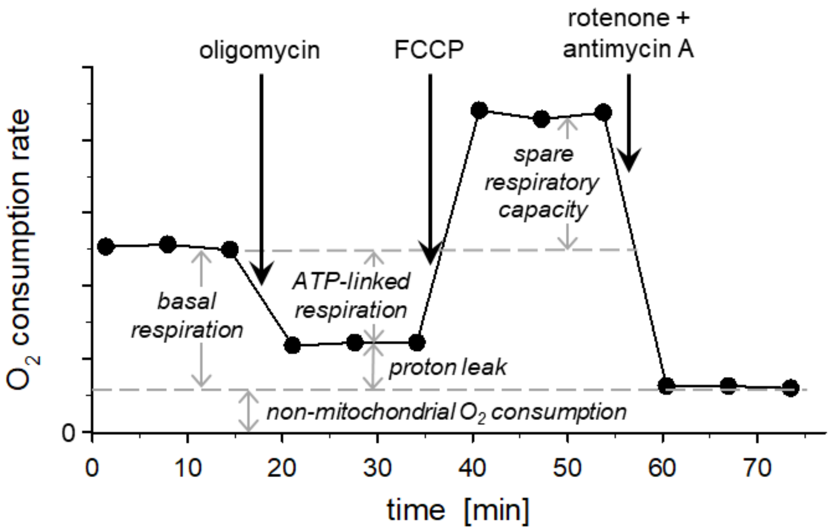


**Fig. S1:** Schematic diagram of the oxygen consumption during the Seahorse Cell Mito Stress Test. The diagram also shows the interpretation of the measurements in the different steps as well as derived (calculated) parameters.

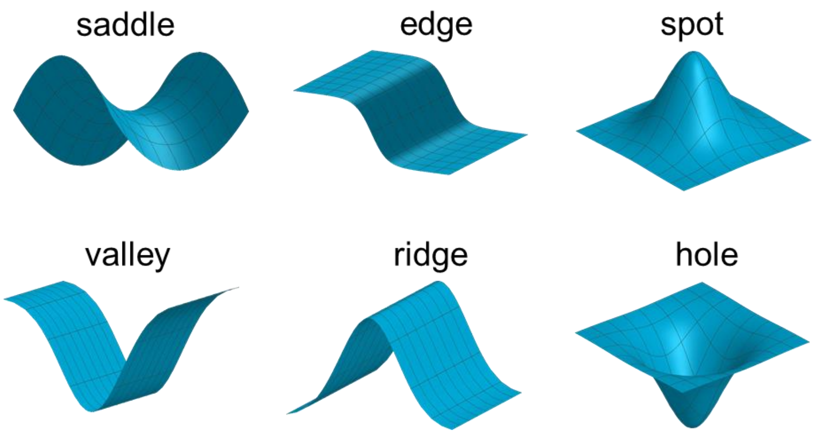


**Fig. S2:** Intensity patterns (e.g., edge, ridges or spots) analyzed by SER texture analysis of the Harmony 4.8 software (PerkinElmer) for the description of the mitochondrial structure.

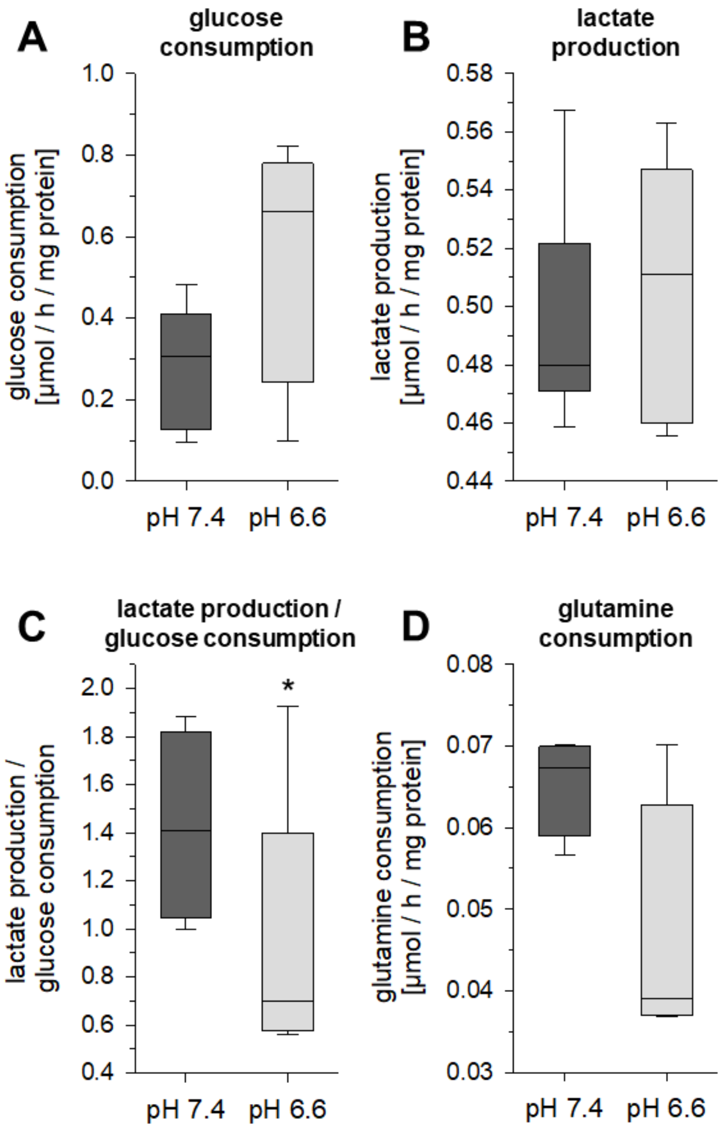


**Fig. S3:** (**A**) Glucose consumption, (**B**) lactate production, (**C**) ratio of lactate production to glucose consumption and (**D**) glutamine consumption in AT1 tumor cells after 3 h under control conditions (pH 7.4) and extracellular acidosis (pH 6.6). n=4-6, (*) $p<0.05$ pH 6.6 *vs.* 7.4.

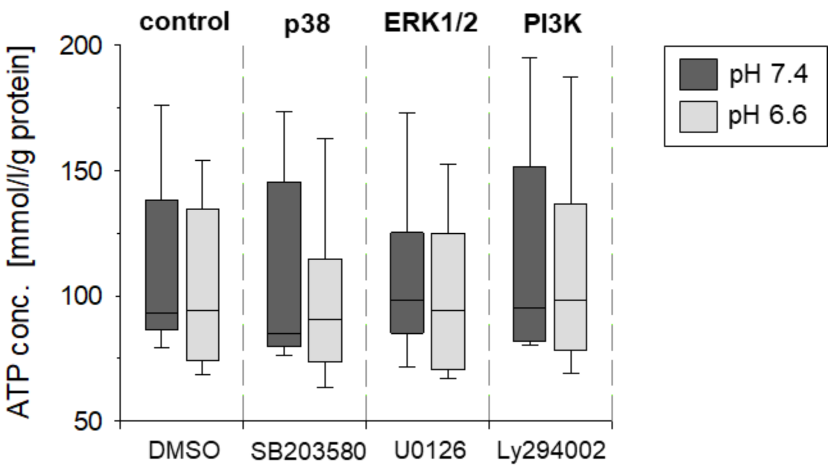


**Fig. S4:** ATP concentration in AT1 tumor cells after 3 h incubation at pH 6.6 or 7.4 and concomitant application of inhibitors of the p38 (SB203580), ERK1/2 (U0126) and PI3K (LY294002) kinases. n=9.

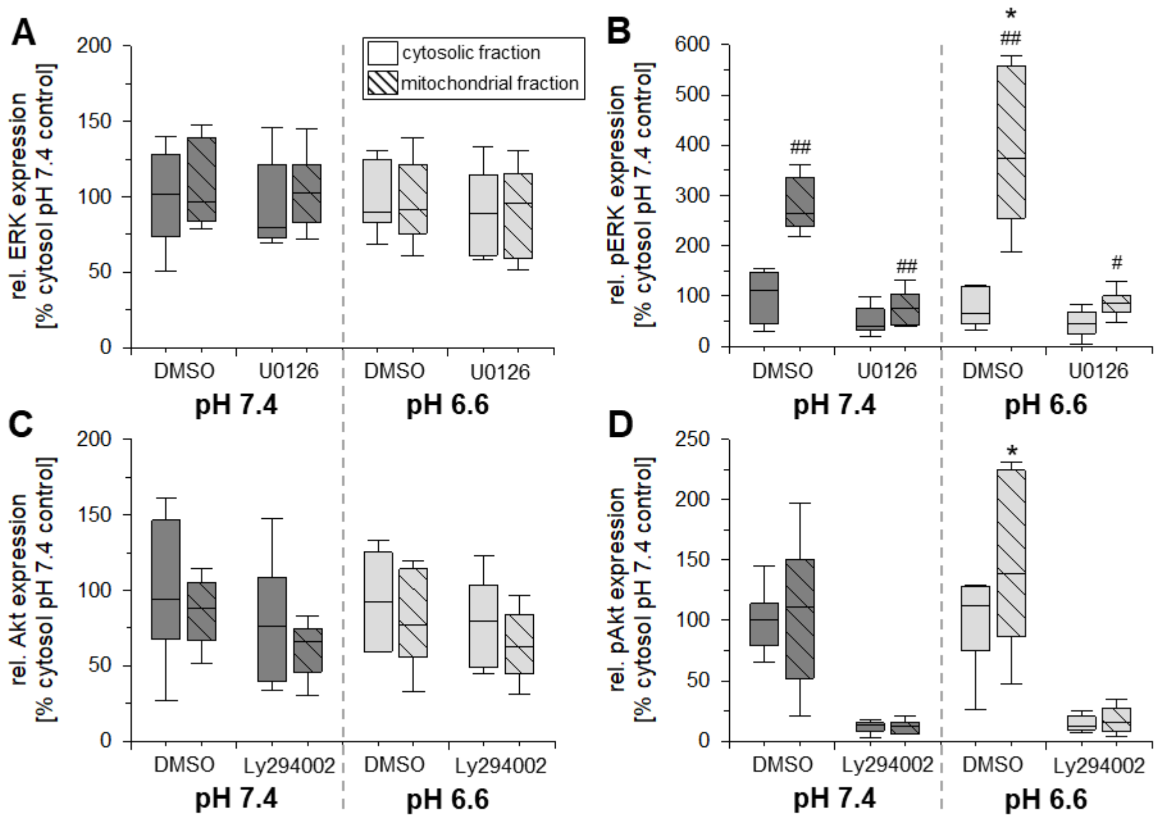


**Fig. S5:** Impact of the extracellular pH (6.6 and 7.4) for 3 h on (**A**) ERK1/2 expression, (**B**) ERK1/2 phosphorylation, (**C**) Akt expression and (**D**) Akt phosphorylation in the cytosolic and the mitochondrial fraction of AT1 tumor cells. Treatment of the cells with inhibitors of ERK1/2 (U0126) or PI3K (Ly294002) as negative controls. n=6, (*) p<0.05 pH 6.6 *vs.* 7.4; (#) p<0.05, (##) p<0.01 cytosolic *vs.* mitochondrial fraction.

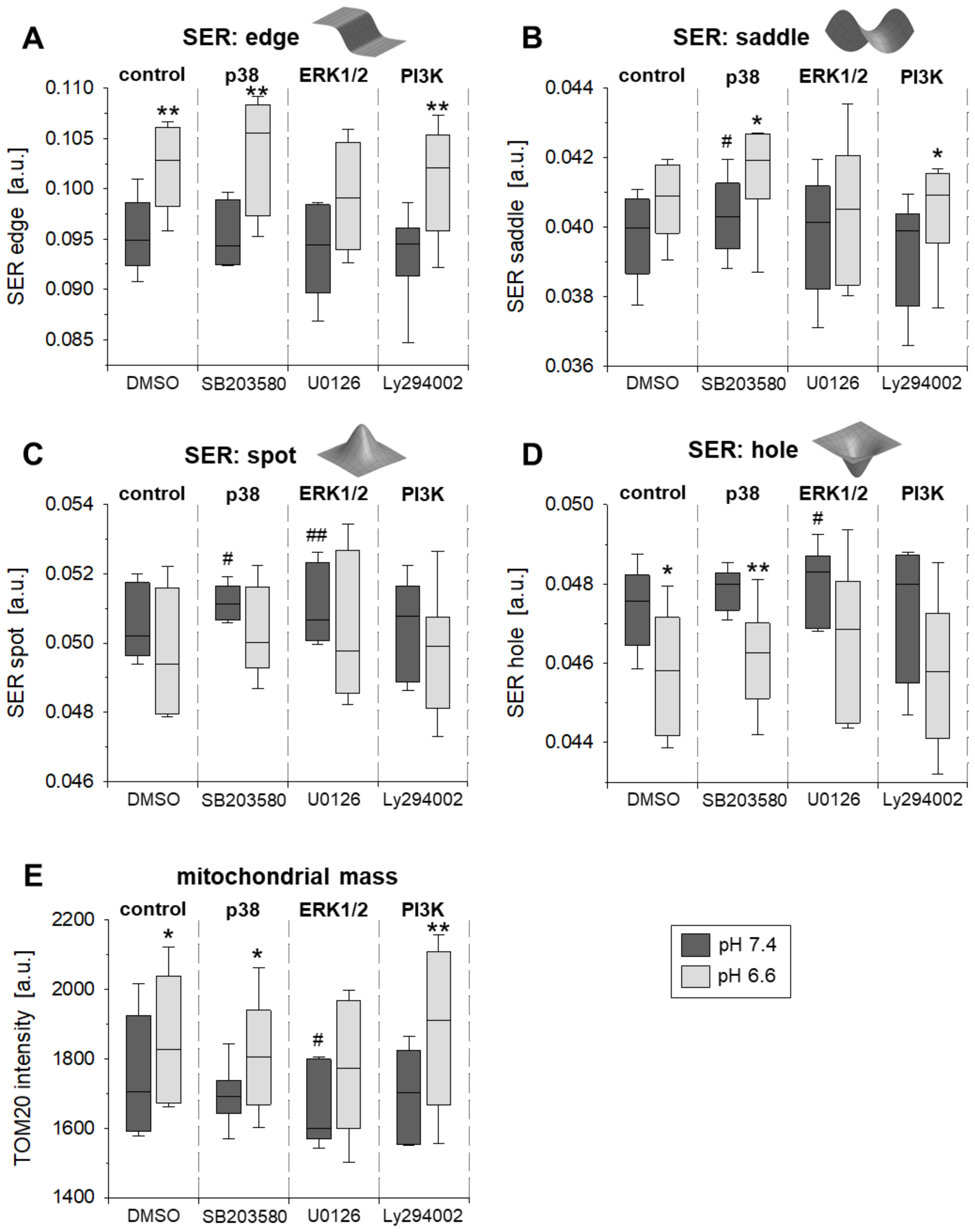


**Fig. S6:** (**A-D**) Parameters of mitochondrial morphology and (**E**) mitochondrial mass in AT1 tumor cells after 3 h incubation at pH 6.6 or 7.4 and concomitant application of inhibitors of the p38 (SB203580), ERK1/2 (U0126) and PI3K (LY294002) kinases. Mitochondrial structure was quantified using the SER (Saddle, Edge, Ridge) texture analysis of cells stained with the mitochondria marker TOM20. The sketches above the graphs show the schematic structures which were quantified by the respective SER parameter. n=6, (*) $p<0.05$, (**) $p<0.01$ pH 6.6 *vs*. 7.4; (#) $p<0.05$, (##) $p<0.01$ *vs*. respective control (DMSO).

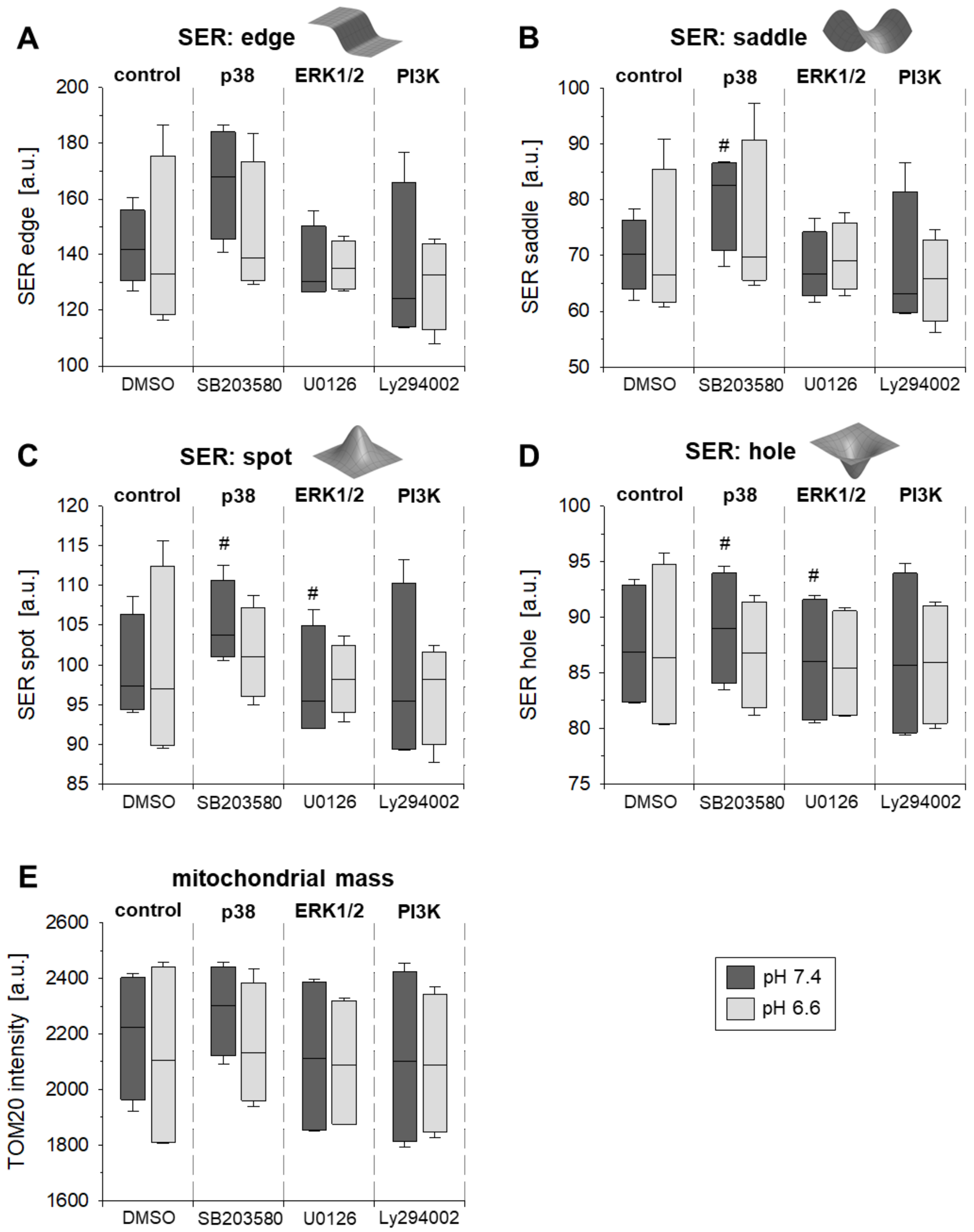


**Fig. S7:** (**A-D**) Parameters of mitochondrial morphology and (**E**) mitochondrial mass in NRKF cells after 3 h incubation at pH 6.6 or 7.4 and concomitant application of inhibitors of the p38 (SB203580), ERK1/2 (U0126) and PI3K (LY294002) kinases. Mitochondrial structure was quantified using the SER (Saddle, Edge, Ridge) texture analysis of cells stained with the mitochondria marker TOM20. The sketches above the graphs show the schematic structures which were quantified by the respective SER parameter. n=6, (*) $p<0.05$, (**) $p<0.01$ pH 6.6 *vs*. 7.4; (#) $p<0.05$, (##) $p<0.01$ *vs*. respective control (DMSO).
